# Ultrasonic signal detection based on Fabry–Perot cavity sensor

**DOI:** 10.1186/s42492-021-00074-0

**Published:** 2021-04-08

**Authors:** Wu Yang, Chonglei Zhang, Jiaqi Zeng, Wei Song

**Affiliations:** Nanophotonics Research Center, Shenzhen University, Shenzhen, 518000 China

**Keywords:** Fabry–Perot microcavity, Double-clad fiber, Acoustic sensor, Endoscopic photoacoustic imaging, Two-photon 3D lithography machine, Full-optical detection

## Abstract

Acoustic/ultrasonic sensors are devices that can convert mechanical energy into electrical signals. The Fabry–Perot cavity is processed on the end face of the double-clad fiber by a two-photon three-dimensional lithography machine. In this study, the outer diameter of the core cladding was 250 μm, the diameter of the core was 9 μm, and the microcavity sensing unit was only 30 μm. It could measure ultrasonic signals with high precision. The characteristics of the proposed ultrasonic sensor were investigated, and its feasibility was proven through experiments. Its design has a small size and can replace a larger ultrasonic detector device for photoacoustic signal detection. The sensor is applicable to the field of biomedical information technology, including medical diagnosis, photoacoustic endoscopy, and photoacoustic imaging.

## Introduction

According to recent reports, an increasing number of chronic diseases are endangering people’s physical and mental health, such as colorectal cancer, liver cancer, stomach cancer, and gastrointestinal cancer [1]. Technologies that enable early detection and timely diagnosis are thus particularly important. Digestive tract diagnosis commonly used in clinical practice is mainly based on *in vitro* imaging detection and endoscopic imaging detection. However, *in vitro* imaging is limited by its resolution and sensitivity; it is often difficult to achieve early detection and diagnosis of small lesions, including nourishing blood vessels (usually small in scale). Medical gastrointestinal endoscopes can directly reach the lesion area for observation; therefore, they can often provide more abundant and accurate lesion details than *in vitro* imaging.

Existing clinical digestive endoscopes mainly include white light endoscopes, fluorescence endoscopes, and ultrasound endoscopes. White light endoscopy and fluorescence endoscopy can provide optical images of the inner surface of the digestive tract, including the superficial blood vessels. Nevertheless, owing to the limited penetration depth, they cannot provide information about the blood vessels deep in the lesion. Although endoscopic ultrasonography has good penetrating ability and can provide deep information, such as the depth of tumor invasion [2], it is difficult to perform fine imaging of tiny early tumors or blood vessels around the tumor because of its low contrast. Clinical endoscopic imaging technology has obvious deficiencies in the early detection and diagnosis of tumors. Thus, development of new endoscopic technologies and devices is urgently needed.

In recent years, photoacoustic biomedical imaging has been studied by many scholars. Photoacoustic imaging is a new type of biomedical imaging method based on the difference in optical absorption and employs ultrasound as the information carrier. Photoacoustic imaging combines the high optical contrast and high penetration depth of the ultrasound to achieve cross-molecule, cell, tissue, and organ imaging [3]. Photoacoustic endoscopic imaging technology, which combines photoacoustic imaging and endoscopic technology, can provide in situ biological tissue structure and function information in the body [4, 5]. Because of its potential for clinical practice, it has become the main research and application development direction of photoacoustic imaging technology.

The photoacoustic endoscopy system integrates optical fibers, ultrasound transducers, mirrors, and microlenses into the tip probe of the endoscope, performs scans by rotating the photoacoustic endoscopic probe, and utilizes the characteristics of the tissue to generate an ultrasound image after absorbing light energy. The transducer performs detection and acquisition, and it reconstructs the optical absorption distribution image of the cavity tissue through an inversion algorithm [6–8]. It has significant clinical application prospects and is thus an increasing focus of researchers.

To realize its clinical application, it is necessary to develop new ultrasonic detection technology. Early photoacoustic imaging technology relies on piezoelectric ceramic ultrasonic transducers, which have property limitations. Its detection bandwidth is not adequately high and the sensitivity is low. In particular, the endoscopic probe is large in volume [9, 10]. The interferometric sensor, which is based on the Fabry–Perot (F–P) cavity, remains valuable to researchers [11, 12]. It can achieve high-sensitivity ultrasonic detection and can simultaneously meet the requirements of a small size and high sensitivity. However, the shape and size of the F–P cavity affect the sensitivity. Therefore, an ultrasonic signal detection system that integrates the F–P flat-cavity sensing unit on the end face of the double-clad fiber (DCF) was developed to achieve miniaturization and high sensitivity. Because the photoacoustic signal belongs to the signal in the ultrasonic range, the sensor unit is integrated on the end face of the DCF with an outer diameter of only 250 μm. The overall size and structure is small, and it has high sensitivity and a large bandwidth. It is expected to be used in endoscopic photoacoustic clinical research.

## Methods and principles

To further miniaturize the endoscopic probe, while engendering a higher sensitivity and larger detection bandwidth, an all-optical detection endoscopic photoacoustic microscopy imaging technology was developed. First, a set of ultrasonic detection devices, which have high integration ability and a small size, were built. They theoretically have the advantages of a higher sensitivity and large bandwidth and can be used for ultrasonic signal detection. The response principle of this ultrasonic sensor was analyzed, and the phase modulation mechanism of the microcavity was assessed.

**Fig. 1:**
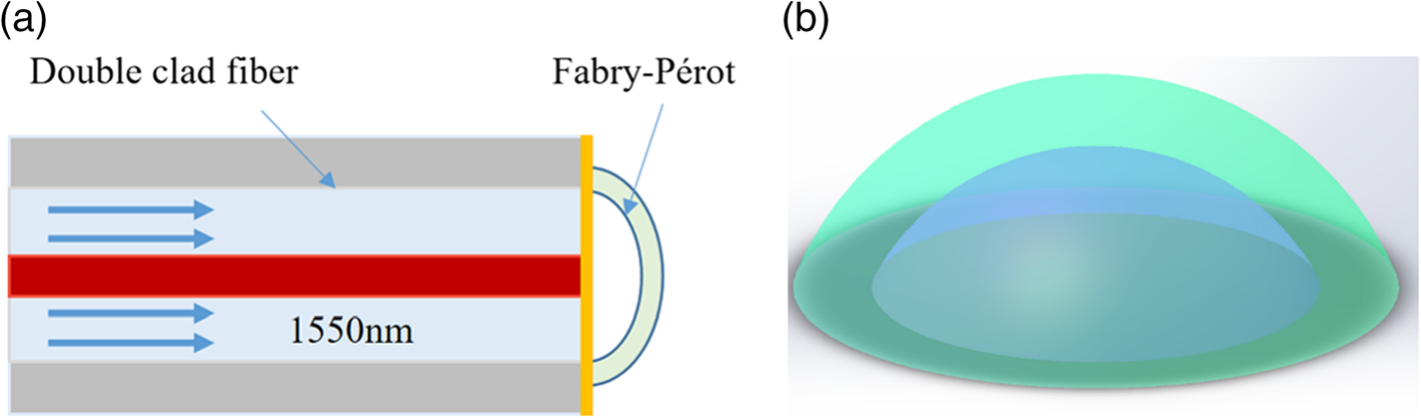
(**a**) Schematic diagram of ultrasound detection based on the F–P cavity. The red area is the fiber core, the light blue area is the first inner cladding of the fiber, and the yellow area is the gold film formed by gold plating on the end face of the fiber. In addition, the arc part is the structure made of photoresist, and the F–P cavity is formed with the end face of the fiber. (**b**) Pattern layout of the F–P cavity using design software to create a model diagram corresponding to the arc-shaped part of (**a**)

To further reduce the size of the detection unit and to detect ultrasonic signals, as shown in Fig. 1, a sensor based on the F–P cavity was designed. When the ultrasonic signal is generated by the ultrasonic transducer and transmitted to the surface of the F–P cavity under the action of the water medium, the ultrasonic wave will modulate the cavity length of the F–P cavity and affect the phase change of the detection light. Multiple interferences in the cavity and the reflected light intensity changes were detected to verify the feasibility of the sensor and its characterization sensitivity.

Because the polymer material has a high optical elastic coefficient and high deformability, the sensitive response can be improved under the action of sound pressure. This optically transparent polymer material is used to process the F–P cavity. It has been shown that the polymer-based fiber-tip F–P cavity ultrasonic hydrophone has a sensitivity and noise equivalent pressure (NEP) comparable to current piezoelectric poly (vinylidene fluoride) (PVDF) ultrasonic sensing devices [13]. Regarding microcavity sensing detection, an investigation and comparison with previous experimental results determined that the flat-concave F–P polymerization cavity can provide better photoacoustic image quality than the flat F–P [14].

**Fig. 2:**
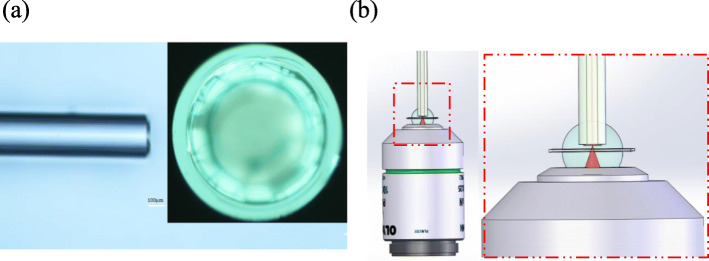
Two-photon lithography using a 3D direct-laser-writing (DLW) system (Photonic Professional GT, Nanoscribe). **a** Left: Cross-section of the processed structure. Upper right: End face of the processed structure. **b** Left: Schematic diagram of the actual machining model. Right: Local enlarged figure in the dotted line box of the left figure

In this study, based on theoretical analysis, the cavity length with a large reflectivity response was selected for a simulation. The cavity length was machined to achieve precision using two-photon 3D photolithography (Photonic Professional GT, Nanoscribe, Germany). A more appropriate flat–concave cavity length and radius of curvature was selected. The cavity length range with high precision was 10–68 μm, and it was processed on the tip of the double-clad fiber by two-photon 3D lithography. The designed F–P cavity structure was intended to ensure precision and efficient batch production, and the two-photon 3D photolithography machine solved the processing problem. It can produce any desired 3D precise structure on the end face of the optical fiber and can choose a photoresist with different refractive indices. This capability engenders the possibility of testing ultrasonic signals in F–P resonators. Therefore, the microcavity is processed and formed by a two-photon three-dimensional lithography machine at one time. It has high precision and can provide highly precise laser scanning to guarantee subsequent high-sensitivity sensor detection. The processing principle is shown in Fig. 2b. The left side of Fig. 2a is the axial view after processing, and the right side is the end face of the microcavity. The difficulty of this method is that the fiber end face must be very clean and flat, which is the key to ensuring mass production in a later stage. The theoretical design minimizes the impact of processing errors on sensitivity and stability.

**Fig. 3:**
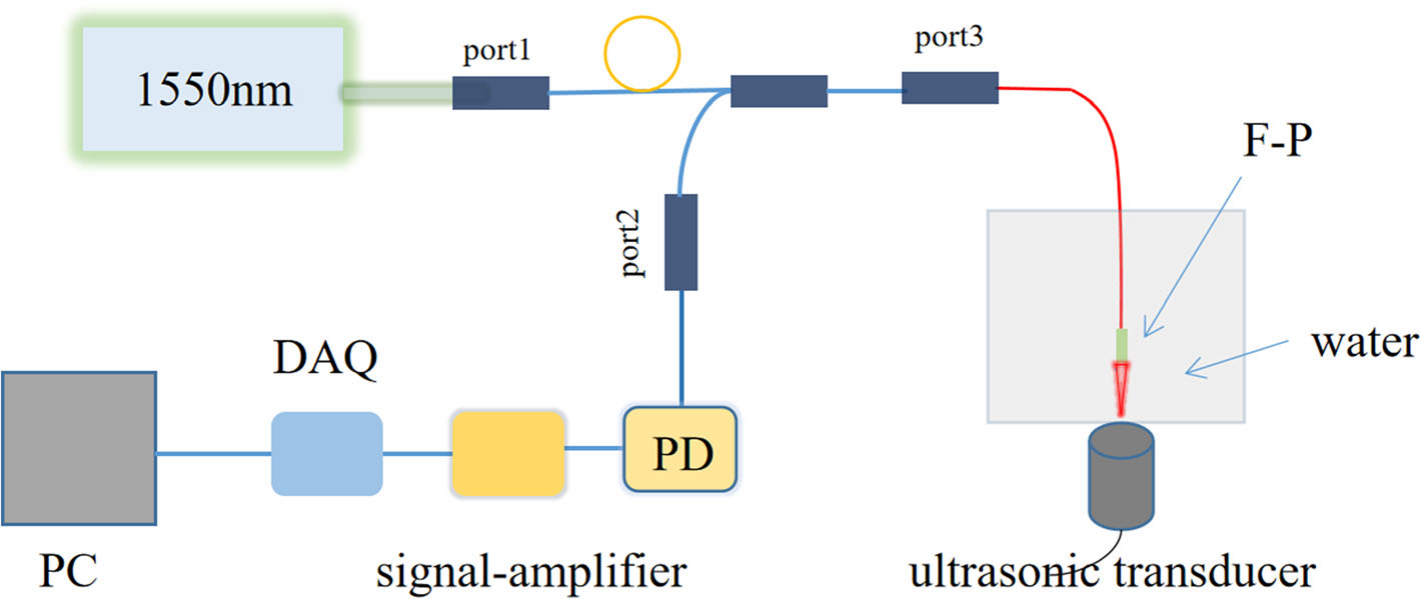
Schematic diagram of the F–P mode system. The 1550 nm fiber laser is connected with toroidal port 1 through a flange device. Interface port 3 is connected with the optical fiber. The optical intensity change returned by the detection light through the F–P cavity interference of the optical fiber is received by the photoelectric detector, and the test results are obtained after amplification and acquisition processing. PD: Photoelectric detector; DAQ: Data collection card; PC: Personal computer

Through the theoretical analysis and simulation, the optical path system was initially established, as shown in Fig. 3. The 1550 nm light is coupled in the double-clad fiber (Thorlabs, DCF13, 1250–1600 nm, Ø105 μm / Ø125 μm cladding), and a 1550 nm light (SM-1550-CEYEL) was used as the interference optical path. A 20 MHz ultrasonic transducer (Olympus, V354, 20 MHz/.25, 1,152,178) was used as the signal source, and deionized water was employed as the coupling medium for ultrasonic signal detection. When the 1550 nm probe light propagates in the F–P cavity, the matched cavity length satisfies the interference of the probe light in the cavity. The ultrasonic pressure wave changes the refractive index of the water on the cavity surface and affects the light interference in the cavity, thereby influencing the reflected brightness changes [15]. The optical sensitivity of the microcavity is defined by a slight change in the reflected light. The round-trip phase of the ideal non-divergent light in the cavity can be expressed as eq. (1). In the case of a fixed wavelength, changes in refractive index and cavity length are two important causes of phase changes, as shown in the equation. In addition, one of the most important primary factors in the detection of acoustic signals is acoustic sensitivity, which is the modulation of the cavity length by pressure dp, as shown in eq. (2). One substitutes eq. (1) into eq. (2) to obtain relational eq. (3), which further exemplifies that the pitch modulation of the microcavity is the main reason for the phase change.

1 $$ \varphi =\frac{4\pi nL}{\lambda } $$

2 $$ \frac{d\varphi}{d p}=\frac{\partial \varphi }{\partial L}\frac{\partial L}{\partial p}+\frac{\partial \varphi }{\partial n}\frac{\partial n}{\partial p} $$

3 $$ \frac{d\varphi}{d p}=\frac{4\pi n}{\lambda}\frac{\partial L}{\partial p}+\frac{4\pi n}{\lambda}\frac{\partial n}{\partial p} $$

Furthermore, one of the measurement indices of the F–P microcavity performance is the high quality (Q) factor, which is defined as the ratio of the energy loss per round trip to the initial energy stored in the cavity. Here, *L* is the cavity spacing of the F–P microcavity length, λ0 denotes the wavelength at resonance, and *k* the fractional power loss coefficient per round trip.

When the F–P cavity design matches the beam, a larger Q value is obtained, as shown in eq. (4). To increase the sensitivity as much as possible by increasing the mirror reflection coefficient of the plano-concave cavity, the light capture time can be prolonged, and the significant number of round trips in the cavity can be increased. This results in a high Q factor and a higher cavity transfer function at the periodic damage [10, 15]. In addition, a coating fixture was designed to coat the end surface of the double-clad fiber with a high-reflection film by thermal evaporation. This helps to increase the adhesion of the F–P cavity on the end face of the fiber and increases the number of round-trips of the light in the cavity, which, once again, results in a higher Q factor and a higher cavity transfer function at the periodic damage [16]. The incident acoustic wave modulates the optical thickness of the F–P cavity, produces an optical phase shift between the optical fields reflected from both sides of the F–P cavity, and produces corresponding reflection intensity modulation.

4 $$ Q=\frac{4\pi L}{\lambda_0k} $$

Another variable that is important to consider is visibility, which is a measure of the depth of the reflectivity peaks in the transfer function [15]. The visibility is defined in eq. (5), where *P*_*max*_ is the maximum power in reflection mode, and *P*_*min*_ is the minimum reflection power at the reflectance minimum of the reflection mode interferometer transfer functions (ITFs). For a non-diverging (collimated) beam, the visibility is 1. The acoustic sensitivity has certain requirements in terms of the choice of cavity thickness for a large acoustic bandwidth. In addition, the mirror material forming the F–P cavity has certain mechanical and optical properties. To increase the sensitivity, it is necessary to build an F–P cavity with higher optical sensitivities. The design of an F–P cavity with higher optical sensitivity could be achieved by increasing the mirror reflectivities [15]. Thus, increasing the reflectivity can effectively enhance the sensitivity.

From eq. (3), the influence of ultrasonic pressure on the cavity length, which in turn affects the change in reflected light intensity, is observed. This thereby affects the sensitivity of the entire probe. Therefore, a high optical sensitivity is achieved when a small modulation in phase * dφ *produces the largest change in the reflected light *dR*. For a non-diverging beam, this can be achieved by increasing the mirror reflectivity to make the slope of the reflectivity peak sharper. The acoustic sensitivity in the F–P cavity is defined as the modulation of the optical phase by a pressure dp. Equation (6) is used to express the overall sensitivity, combined with the acoustic sensitivity expression in eq. (2), and obtain the overall expression of the sensitivity of eq. (7), where *R* is the reflectivity and *E* is Young’s modulus [17].

5 $$ V=\frac{P_{max}-{P}_{min}}{P_{\mathrm{m} ax}+{P}_{min}} $$

6 $$ s=\frac{d R}{d p}=\frac{d R}{d\varphi}\frac{d\varphi}{d p} $$

7 $$ s=\frac{d R}{d\varphi}\frac{4\pi n}{\lambda}\frac{L}{E} $$

## Results

**Fig. 4:**
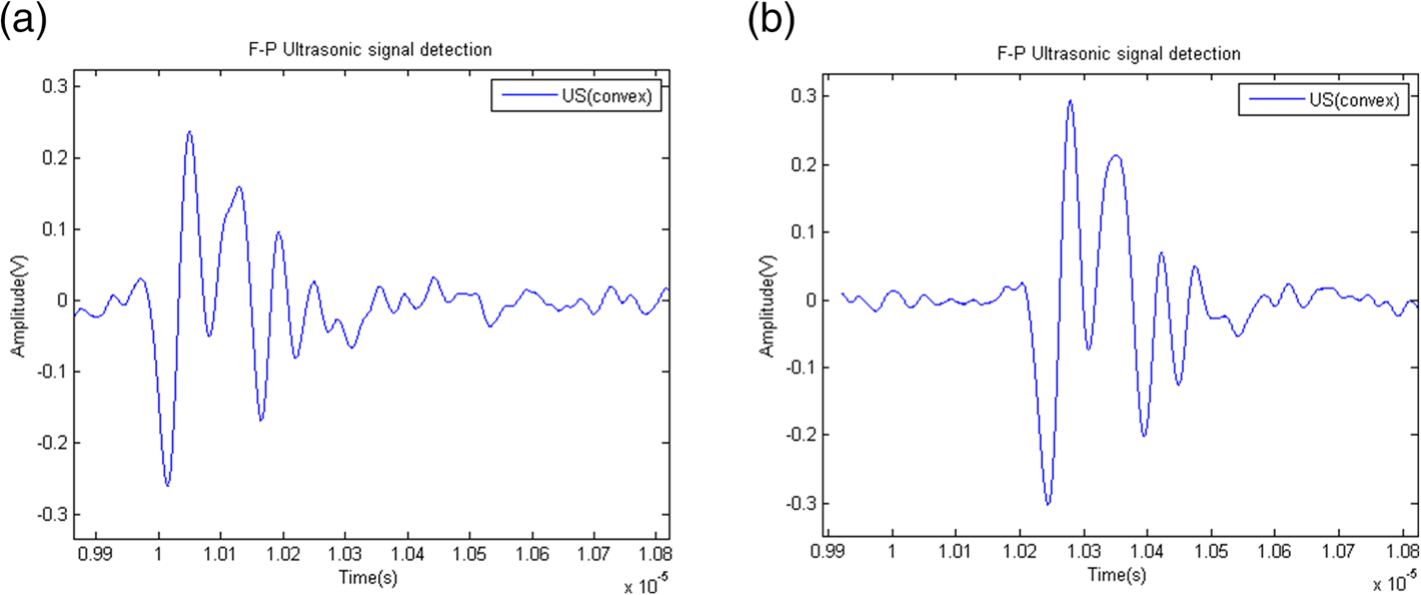
Frequency response calibration: F–P measured time-domain signal. The fiber is placed vertically and inserted into the tank, as shown in Fig. 3. The bottom of the tank is a layer of transparent film, and the bottom of the cling film is the ultrasonic transducer, which is vertically upward relative to the optical fiber. The distance between the end face of the optical fiber and the transducer is approximately 1 mm. The signal is measured by adjusting the distance and coupling angle between the transducer and the optical fiber

In theory, the actual processing technology was exemplified and analyzed, while considering the related issues such as the system stability. Moreover, the optical path of the system was optimized many times. Considering the experimental test problem, the ultrasonic transducer was used as the ultrasonic signal source, and the measured acoustic signal was nearly 13 Pa. At the present, we remain committed to advancing this research to realize higher sensitivity. Through the optimized design of the F–P structure, the theoretical sensitivity could reach several tens of Pa to several Pa. Thus, biological tissue imaging is expected to produce very good results. For this study, a flat cavity with a cavity length of 30 μm and a radius of curvature of 680 was used. The ultrasonic source used was 20 MHz. The test medium was deionized water. The test results are shown in Fig. 4.

For convenience of testing, the ultrasonic transducer was assembled on the 3D translation device and fixed the position of the sink. This avoided the direct movement of the sensor causing sensor noise disturbance. When the ultrasonic transducer was moved, the signal changes could be measured, as shown in the two signal diagrams on the left and right of Fig. 4. The measured result has a sensitivity of approximately 13 Pa (the peak-to-peak of the signal value divided by the root mean square of the noise) and a bandwidth of nearly 20 MHz. For the treatment methods, previously reported studies [18] were referenced. The Q factor was thus estimated to be approximately 3246 [=1550 × 2π/3]. The peak output voltage measured by the F–P sensor was 302 mV under a 107-kPa acoustic pressure. Thus, the sensitivity of the F–P sensor was determined to be approximately 2.82 mV/kPa. Meanwhile, considering that the actual detection sensitivity was not the expected result, the Q value of the analysis cavity was theoretically greater than that of the planar microcavity [10]. An improved concave microcavity design in one step will continue to be developed. Our research on the integration of different microcavity structures on the end face of double-clad fibers will also be expanding.

## Conclusions

Optical ultrasonic inspection technology may offer the prospect of overcoming the limitations described above. Compared with piezoelectric receivers, optical ultrasonic inspection technology can provide reverse mode detection and greatly reduce the size of the element. In principle, it can reach the optical diffraction limit of a few microns [19]. In summary, the feasibility of detecting ultrasonic signals based on the microcavity detection ultrasonic device was herein verified. The proposed design can be expected to replace the larger piezoelectric ceramic detector [20]. It has the advantages of a small size, high sensitivity, and mass production capability.

## Discussion

This present work is intended to improve the signal-to-noise ratio and signal stability of ultrasonic signals. After this objective is achieved, the advantages of double-cladding fiber will be leveraged to simultaneously couple the excitation light and detection light into the double-cladding fiber for all-optical endoscopic detection. The sensing unit can be further optimized and used as a photoacoustic endoscopic probe for clinical trials of the digestive tract. It can additionally be employed for photoacoustic endoscopic imaging research. It has the advantages of a low cost, no ionization, no radiation, and high-resolution real-time imaging of the human body. In particular, imaging the tissues in the cavity and plaques in the blood vessels can improve the early diagnosis rate of tumors. This type of sensor based on microcavity detection offers the benefits of an intrinsic type, all-fiber integration, small size, and high sensitivity. The designed micro-cavity detection ultrasound unit is expected to be used in all-optical endoscopic detection. It will realize non-destructive detection and a more miniaturized sensor probe to achieve a greater number of functions and to more effectively serve clinicians.

## Data Availability

All data analyzed during this study are included in this published article.

## Abbreviations

F–P: Fabry–Perot
DCF: Double-clad fiber
PVDF: Poly (vinylidene fluoride)
NEP: Noise equivalent pressure
Q: Quality factor
DLW: Direct-laser writing
PC: Personal computer
DAQ: Data acquisition
PD: Photoelectric detector
ITF: Interferometer transfer function
