# Correction to: Ultrasonic signal detection based on Fabry–Perot cavity sensor

**DOI:** 10.1186/s42492-021-00079-9

**Published:** 2021-05-10

**Authors:** Wu Yang, Chonglei Zhang, Jiaqi Zeng, Wei Song

**Affiliations:** Nanophotonics Research Center, Shenzhen University, Shenzhen, 518000 China

**Correction to: Vis Comput Ind Biomed Art 4, 8 (2021)**

**https://doi.org/10.1186/s42492-021-00074-0**

Following the publication of the original article [1], it was noted that due to a typesetting error the Fig. 1(b) was missing in the web version.

The complete Fig. 1 has been included in this correction, and the original article has been corrected.

**Fig. 1:**
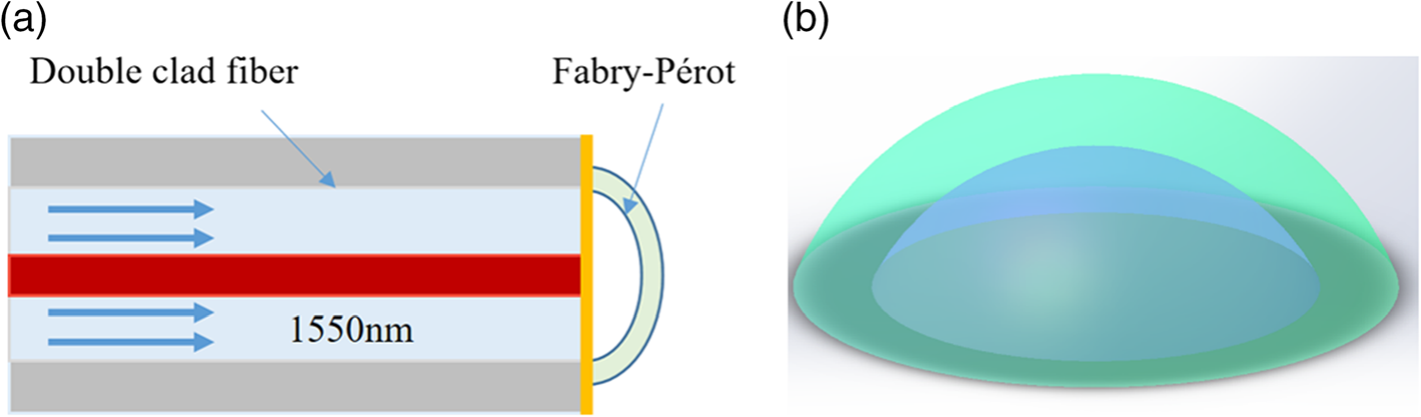
(**a**) Schematic diagram of ultrasound detection based on the F–P cavity. The red area is the fiber core, the light blue area is the first inner cladding of the fiber, and the yellow area is the gold film formed by gold plating on the end face of the fiber. In addition, the arc part is the structure made of photoresist, and the F–P cavity is formed with the end face of the fiber. (**b**) Pattern layout of the F–P cavity using design software to create a model diagram corresponding to the arc-shaped part of (**a**)
